# Determinants of Health Inequalities in Iran and Saudi Arabia: A Systematic Review of the Sleep Literature

**DOI:** 10.3390/clockssleep5010013

**Published:** 2023-03-22

**Authors:** Comsar Ndiaye, Yosr Ayedi, Faustin Armel Etindele Sosso

**Affiliations:** 1 Graduate School of Public Health, Paris-Saclay University, 91190 Paris, France; 2 Department of Epidemiology and Biostatistics, Abderrahmane Mami University Hospital, Ariana 2080, Tunisia; 3 Department of Global Health and Ecoepidemiology, Redavi Institute, Montréal, QC H4J 1C5, Canada

**Keywords:** Iran, Saudi Arabia, socioeconomic status, sleep health inequalities, systematic review

## Abstract

Sleep health inequalities represent an increasing public health concern. Among multiple determinants affecting sleep health, there is people’s socioeconomic status (SES), and no systematic review on the relationship between SES and sleep health has been previously conducted in Iran and Saudi Arabia. Following the Prisma protocol, ten articles were selected. Findings revealed that the combined number of participants was N = 37,455 participants, including 73.23% of children and adolescents (*n* = 27,670) and 26.77% of adults (*n* = 10,026). The smallest sample was N = 715 and the larger was N = 13,486. In all these studies, sleep variables were assessed using self-reported questionnaires. The studies conducted in Iran assessed the risk of obstructive sleep apnea (OSA), while those in Saudi Arabia were interested in sleep duration, nap time, bedtime, rise time and insomnia. The studies performed on adult populations in Iran and Saudi Arabia concluded that there is no significant association between SES determinants and sleep components in adult populations. One study in Iran found a significant association between parent’s low SES and children and adolescent insomnia; and one study in Saudi Arabia found a significant association between the father’s education and the longer sleep duration of their children. More longitudinal studies are necessary to establish a causal relationship between public health policies and sleep health inequalities. An extension of the investigation to more sleep disturbances is required to cover the entirety of sleep health inequalities in Iran and Saudi Arabia.

## 1. Introduction

Health inequalities are associated with economic and societal impact on every government, regardless of GDP and the global well-being of an entire population [1,2,3,4,5,6,7,8]. Health inequalities can be observed with the cardiovascular system [9], respiratory system [10] or sleep mechanisms [1,2]. Sleep is a multifactorial process that may be disturbed by a wide range of environmental, sociological, physiological stimuli and socioeconomic status (SES) [4,5,11]. The concept of sleep health provides a positive holistic framing of multiple sleep characteristics, including sleep duration, continuity, timing, alertness and satisfaction [4]. Sleep health inequalities represent a public health concern similar to public health issues previously reported for cardiovascular, mental health and metabolic diseases [12], while SES is a latent multidimensional concept of an individual’s economic and socioecological situation, also associated to public health inequalities at different levels such as community or country [5,11]. Thus, sleep health disparity is a complex assessment of socio-ideological and theoretical constructs measured in a variety of ways, usually considering several determinants such as employment, income, education, occupation and social position [3,11,12,13].

An extensive screening of the empirical literature revealed that no systematic review on the relationship between SES and sleep health has been previously conducted in Iran and Saudi Arabia. It is pertinent to understand whether public health inequalities in terms of sleep disturbances that are observed elsewhere are the same as in these two important countries, especially considering the crossover of different continents and different ethnocultural populations. This systematic review aims to document the determinants of sleep health inequalities in Iran and Saudi Arabia.

## 2. Results

### 2.1. Characteristics of Studies Selected

***Descriptive synthesis.*** Ten articles [14,15,16,17,18,19,20,21,22,23] were included in the final sample (Table 1). They were all cross-sectional studies and published between 2006 and 2019. Among these studies, 50% (*n* = 5) were performed in Saudi Arabia and 50% (*n* = 5) in Iran. The combined number of participants was N = 39,550 participants, composed of 69.35% children and adolescents (*n* = 27,429, mean age = 9.8 years old) and 30.65% adults (*n* = 12,121, mean age = 46.3 years old). The smallest sample was N = 715 and the largest were N = 13,486. The socioeconomic indicators used were perceived SES/composite indices, education, income, marital status and employment/occupation. In all the studies, sleep variables were reported using self-reported questionnaires. The studies conducted in Iran assessed principally the risk of obstructive sleep apnea (OSA), while those in Saudi Arabia were interested in sleep duration, nap time, bedtime, rise time and insomnia.

***Sleep fragmentation.*** Four cross-sectional studies [18,21,22,23] have studied the association between education (not educated, high school or less and university) with the outcome of the risk of obstructive sleep apnea disorder and insomnia. Amra et al. [18] (adults from the general population aged from 18 to 70 years old) and Seyedmehdi et al. [21] (staff employees at a hospital with mean age at 33.5 years old +/− 7.65) were rated as good quality articles and used the Berlin Questionnaire. Foroughi et al. [22] (adults from the general population with mean age at 40.88 +/− 15.4) had used the Stop Bang. It was rated as a poor-quality study. Ahmed et al. [23] used the ICD-2 instrument to assess insomnia symptoms. No significant result in the multivariate analysis was found in all three articles.

***Sleep latency.*** One cross-sectional study [23] was performed on adults (mean age at 42.3 years +/− 15.5). It had used the ICD-2 criteria to test the association between education and having insomnia, defined by the presence of any of the following symptoms: (1) early morning awakening, (2) difficulty initiating sleep, (3) frequent awakening, (4) feeling tired and (5) sleep loss affects mood. This study was rated as fair. Overall, uneducated people had more risk of insomnia than those attended university. Those who attended high school had a lower risk of insomnia compared to those who attended university. This study concluded that insomnia is most prevalent among females and the elderly population.

***Total sleep time.*** The cross-sectional study of Althakafi et al. [16] had been conducted using an online questionnaire. The mean age of participants was 21.8 years +/− 10.7. It was rated as having a good quality. It had studied the association between education and the duration of sleep and found no significant association.

### 2.2. Determinants of Sleep Health Inequalities in Iran

Four cross-sectional studies assessed the relationship between SES and subjective sleep measurement. One of these was conducted on children and adolescents (mean age = 12.47 years old) and the others with adults.

For the study conducted on children and adolescents, SES was measured by a SES score summarized by parents’ education and occupation, possessing a private car, school type (public/private), home type (private/rented) and having a personal computer at home. Sleep variables were sleep duration and insomnia assessed by a non-specific questionnaire administered both to parents and children by interviewers.

For those conducted on adults, the SES measurement was the educational level. Sleep variables were risks for obstructive sleep apnea assessed by the self-reported Berlin questionnaire in two studies and the Stop Bang questionnaire in one study. One of these studies assessed, additionally, the quality of sleep summarized by difficulty falling asleep, difficulty staying asleep and waking up too early.

Overall, there was no association between SES and sleep variables. Specifically, only the study conducted in children and adolescents reported an association between low socioeconomic conditions of parents and insomnia. The association between sleep duration and SES was not assessed in this study. In adults, no association between educational level and self-reported risk of OSA was found.

### 2.3. Determinants of Sleep Health Inequalities in Saudi Arabia

Among the five studies conducted in Saudi Arabia, two were conducted on children, one on adolescents and two on adults. For those conducted on children, SES was measured by the educational level of parents, job status of mothers and family income. For the one conducted on adolescents, the SES measurement was self-reported perceived socio-economic status. For the last two articles on adults, SES was measured by educational level, marital status, occupation and job status. Sleep variables were principally night sleep duration. In one study conducted on children nap duration, bedtime and rise time were also measured. For the studies conducted on adolescents and adults, sleep duration was used to characterize sleep deprivation which was defined by sleeping less than 7 h daily.

Overall, no association was found between SES and sleep duration or sleep deprivation. Only one study conducted on children reported that a high educational level of fathers was associated with longer sleep duration.

## 3. Discussion

### 3.1. Summary of Findings

All these ten studies were cross-sectional studies including a total number of 39,550 participants, an average of 55.94% of women in Iran and 52.92% of women in Saudi Arabia. The majority of participants were children and adolescents (N = 27,670, 73.23%). The SES indicators used were perceived SES/composite indices, education, income and employment/occupation. Sleep components were reported using self-reported questionnaires. Studies conducted in Iran assessed principally the risk of obstructive sleep apnea while those in Saudi Arabia were interested in sleep duration, and additionally in nap time, bedtime, rise time and insomnia. The studies performed on adult populations in Iran and Saudi Arabia concluded that there is no significant association between SES and sleep components in adult populations. One study in Iran found a significant association between the parent’s low SES and children and adolescent insomnia, and one study in Saudi Arabia found a significant association between the father’s education and longer sleep duration of their children.

### 3.2. Relation with Current Knowledge

The novelty and the significance of this work is the epidemiological investigation of health determinants in a world region not well documented in the field of health inequities and sleep science. The current findings in this research are mitigated because they confirm an association between the parent’s SES and sleep disturbances, but they reported no association between SES and sleep health for adult populations. The majority of studies investigating sleep health inequalities found, until now, an important trend in favor of an association between SES and sleep inequalities [2,3,12,24,25,26,27,28]. Most of them seem to support the hypothesis stating that low SES is associated with an increase in sleep disturbances for children, adolescents and adults [1,10,25,29,30,31,32], while a moderate or a high SES is associated with a decrease in sleep disturbances for the same sub-groups of the general population [2,4,10,11,12]. One reason explaining this empirical discrepancy is the small quantity of research on sleep health inequalities. As revealed by this review, the empirical literature included peer review papers published from 2006 to 2019. It seems relatively new compared to sleep science or public health research related to sleep in other countries, where a few studies on the same subject were published almost twenty years before the first study included in this systematic review [33,34]. Another potential reason explaining the current findings is the experimental design, which is essentially cross-sectional. It is commonly admitted and demonstrated that a cross-sectional study is one of the best experimental designs to rigorously collect data and quickly release pertinent results on a subject [35]. At the same time, it is missing a lot of causal information and correlation, which is very often available with longitudinal study design [36]. It is difficult to establish the causal effects of SES on sleep disturbances in these circumstances. The third reason hypothesized is the incomplete conceptualization of SES in this public health literature. The majority of these articles employed education as a key component to assess the health inequalities of their sample. Education alone cannot capture the complexity of socioeconomic determinants of health inequalities in general and sleep health inequalities in particular [5,6,11,12,37]. It is important to analyze other indicators such as income and employment to understand how far sleep disturbances are important for public health in the countries concerned.

### 3.3. Recommendations for Future Research

*The first recommendation* is to perform longitudinal studies to capture the effect of time in the relationship between SES, public health and sleep. Life conditions are markers of health status variance in time and with changes in demography and lifestyle in certain subgroups of the general population, it might be interesting to observe how sleep patterns evolve negatively or positively for future public health policies.

*The second recommendation* is to use a quantitative measure of sleep components to collect unbiased data. Using actigraphy or polysomnography will add an additional layer of evidence in support of future appropriate public health programs.

*The third recommendation* is to extend the scope of future research by investigating other sleep disorders. The current literature in Iran and Saudi Arabia focuses on insomnia and OSA, even if a few general sleep parameters such as sleep quality and sleep duration were also explored. Sleep science is very wide and multidisciplinary. An integration of different conceptualizations and theoretical models developed by sleep scientists based on inputs from sociology, psychology or health economics are a few examples of possible actions to improve future research and clinical practice.

### 3.4. Limitations of This Review

There was no objective sleep measurement in all studies included. The self-assessment of sleep outcomes is very often used in cross-sectional studies, but it was recently demonstrated that the SES influence on health can be observed with quantitative methods [1,2,38]. So, future studies in this region should consider moving forward the knowledge by using an objective measure of sleep to verify trends reported by local authors. There are also a lot of sleep disorders not studied, while a few such as OSA can be linked to other sleep disorders such as excessive daytime sleepiness [8,39,40], and thus have to be studied. Finally, the heterogeneity of SES involved a deep consideration of the sociological approaches and psychological theories in biomedical research, in order to be able to accurately capture this complex concept.

## 4. Methods

### 4.1. Literature Search

**Figure 1:**
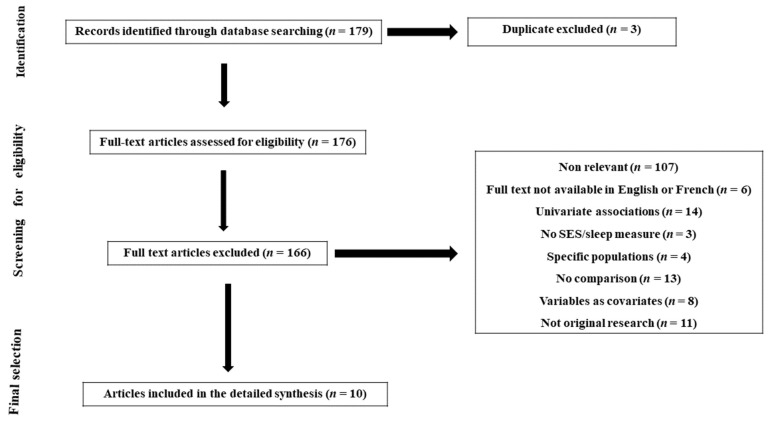
Prisma flowchart of study selection process.

The relevant citations for this review were identified by searching the databases Web of Science, PubMed/Medline and Google Scholar between January 2000 and July 2022. A combination of the search terms “socioeconomic”, “socio-economic”, “social position”, “social class”, “socioeconomic position”, “determinant*”, “health inequalities”, “sleep”, “sleep disorders”, “sleep disturbances”, “sleep complains”, “sleep outcome”, “sleep health”, “Iran*”, “Saudi Arabia” was used. All included articles were identified following the PRISMA guidelines detailed in Figure 1 [41].

### 4.2. Inclusion and Excusion Criteria

Empirical studies were defined as being of any design (cross-sectional, retrospective or longitudinal) that evaluated humans of any age, gender or race/ethnicity from the general populations of Iran and Saudi Arabia. The article should include an objective or a subjective measure of SES, such as education, income, assets, occupation, employment status, perceived SES or self-reported by participants. Indirect measures of SES, such as area deprivation or social class, were also included when individual data were not available. For studies examining children or adolescents, perceived family SES measures such as parental education, parental profession or annual household income were used instead. Studies were excluded based on the following criteria: (1) they were interventional trials, reviews or meta-analyses, case series or case reports, commentary, editorial or did not present original research; (2) they were not written in English, French or Arabic; (3) the full text was not accessible; (4) authors/researchers recruited participants that already presented specific conditions at the beginning of the study (for example, pregnant women or people with chronic sleep disturbances, those taking medication such as sleep aid, etc.); (5) they did not provide statistical significance in cases where either SES or sleep were evaluated as covariates or mediators.

## Figures and Tables

**Table 1:** Characteristics of included studies investigating determinants of sleep health inequalities in Saudi Arabia and Iran.

Study	Study Design	Population	% Women	Age (Mean § SD or Range)	Sample Size	SES Indicators	Sleep Measures	Statistically Significant Findings	Conclusions
**Bahammam, 2006**	Cross-sectional	Children recruited from regular governmental elementary schools in all grades (1 to 6) in Riyadh	49.5	6–13 y	1012	Educational level of parents (illiteracy < or = high school, graduate or postgraduate) Occupation of mothers (housewives, teachers, medical profession, administrators)	Self-administrated questionnaires addressed to parents to assess: Night-time sleep duration = TST Daytime nap duration Bedtime Rise time All measured both during weekdays and weekends	Bedtime for students whose mothers were illiterate or had elementary school education was significantly delayed (21.7 ± 1.2 h and 21.6 ± 1.3 h, respectively), compared to students whose mothers had university or postgraduate education (21.4 ± 1.4 h, *p* = 0.002) TST during weekdays was significantly more in students whose mothers had high school or higher education (8.5–8.7 h) compared to students whose mothers were illiterate or had elementary school education (8.1 ± 1.2 h and 8.2 ± 1.3 h, respectively, *p* < 0.001). TST during weekdays was significantly more in students whose mothers were working (8.6 ± 1.1 h) compared to students whose mothers were housewives (8.4 ± 1.2 h, *p* = 0.005	Children whose mothers had high school education or higher had longer night-time sleep duration compared to children whose mothers had lower education. No association for TST and job status after controlling the cofounders. No relation could be found between father’s level of education or job status and student’s sleep
**Ahmed, 2017**	Cross-sectional	Adults recruited from blood bank donors, preemployment clinics, and KAMC employees	40.7	42.4 ± 15.5 y	2095	Marital status (single, married, widow, and divorced) Education levels (non-educated, elementary, intermediate, high school, and university) Occupation (employed, housewife, students, and unemployed)	Arabic version of the ICSD-2 instrument to assess insomnia symptoms	The gender-adjusted prevalence of insomnia was higher for females, 88.7% (95% CI = 86.4–90.7%) than for males, 70.4% (95% CI = 67.8–72.9%), *p*-value = 0.001. The age-adjusted prevalence of insomnia was higher for the elderly, 93.7% (95% CI = 90.6–96.0%) than for the middle aged, 79.8% (95% CI = 77.4–82.1%), or for the young group, 64.2% (95% CI = 59.9–68.4%), *p*-value = 0.001	Insomnia is most prevalent among Saudi females and the elderly Saudi population.
**Al hazza, 2019**	Cross-sectional	Children enrolled in elementary school in Riyadh	50.2	5.9–13.4 y	1051	Educational level of parents Family income	Sleep duration in weekdays and weekends reported by parents using a questionnaire Insufficient sleep defined as sleeping less than 9 h per night, according to the definition of the National Sleep Foundation for school-age children 6–13 years.	Father education (<high school = ref): high school, college degree, postgraduate degree associated with higher sleep duration, respectively, with Ora of 1.80, 2.01, 3 and *p* < 0.011, *p* < 0.07, *p* < 0.03	Higher father educational levels associated with longer sleep duration. no significant association was found mother education after controlling for cofounders No association for family income, number of family members in the house
**Al althakafi, 2019**	Cross-sectional	Adults randomly recruited in known social media platforms using Google form in Saudi Arabia	63	21.8 ± 10.7 y	805	Educational level (primary, intermediate, secondary, university or more) Job status (students, employee, not working, retired)	Self-reported sleep duration using online questionnaire	In univariate analysis, 80% of those who had primary educational level sleep for <7 h daily compared with 51.4% of university graduated participants with statically significance (*p* < 0.045) 80.8% of retired population sleep for <7 h daily compared with 44.1% of students (*p* < 0.001)	No association found between SES and Sleep duration after controlling for cofounders
**Nasim, 2019**	Cross-sectional	Adolescents recruited in intermediate and secondary schools in Saudi Arabia	49	10–19 y	12,121	Self reported perceived socio-economic status	Sleep deprivation (SD) defined as less than 7 h sleep duration per day assessed by self-report questionnaire: Self-reported sleep duration during weekdays Sleep duration during weekend Number of days per week with nap time	Adolescents who considered themselves to be “poorer than others” in socioeconomic status (OR 1.51, CI 1.28–1.78) Adolescents who reported eating fewer than three meals per day (OR 1.17, CI 1.09–1.27) were also more likely to report SD	Adolescents with poorer perceived SES were more likely to report SD
**Amra, 2011**	Cross-sectional	Randomly selected sample of adults living in urban cities in Ispaphan, Iran	53.3	15–92 y	3529	Educational level (junior high school or less, high school, university)	Risk of OSA diagnosed by self-report Berlin questionnaire	Low educational level was associated to high risk of OSA in chi-square test (*p* < 0.001)	No association between SES and risk of OSA after controlling for cofounders
**Hassani, 2015**	Cross-sectional	Adults working at a hospital in Iran	62.8	33.51 y (SD = 7.65)	715	Educational level (graduate or postgraduate vs. undergraduate)	Risk for OSA syndrome regarding the Berlin questionnaire High-risk for OSA defined by positive scores in two out of the three parts Quality of sleep (difficulty falling asleep, difficulty staying asleep and waking up too early)	406 (56.8%) worked the night shift 367 (51.3%) reported less than four hours of sleep at night during last week 218 (30.5%) had difficulty falling asleep, 233 (32.6%) could not stay asleep and 301 (42.1%) woke up too early As for the probability of sleep apnea, 49 (6.9%) were reported to be highly probable Prevalence of snoring was reported to be 12.6% (90) High educational level was associated with risk for occupational accidents (OR = 1.454 CI = 1.005–2.104, *p* = 0.047)	In hospital staff, high educational level and high risk of OSA are associated to an increased risk for occupational accidents
**Heshmat, 2016**	Cross-sectional	Children and adolescents randomly selected in 48 provinces in Iran	49.2	12.47 ± 3.36 y	13,486	SES score summarized by parents’ education, occupation, possessing a private car, their school type (public/private), home type (private/rented) and having a personal computer at home The calculated score was categorized into tertiles to define SES levels. The 1st tertile was considered ‘low’, and the second and third ones as ‘middle’ and ‘high’ SES, respectively.	Sleep duration and Insomnia assessed by questionnaire administered to parents and children by trained interviewers	In the multivariate model, the OR for insomnia was lower in students with a high SES (OR = 0.79; 95% CI 0.68 to 0.92) compared with low SES students Sleep duration >8 was lower in children living in urban areas (75.03% vs. 81.89%) than those living in rural area	A low SES is associated with insomnia in children and adolescents
**Seyedmehdi, 2016**	Cross-sectional	Staff employees of a hospital in Tehran, Iran	62.8	33.51 ± 7.65 y	715	Education (undergraduate vs. graduate or postgraduate)	OSA risk (Berlin questionnaire, high vs. low risk)	The association between the risk of sleep apnea and night sleep less than 4 h (OR = 2.273, CI = 1.142–4.523, *p*-value = 0.019) and difficulty in staying asleep (OR = 2.515, CI = 1.258–5.031, *p* value = 0.009) showed to be statistically significant	There was no association between education and OSA risk
**Foroughi, 2017**	Cross-sectional	A random sample of adults living in Tehran, Iran	51.6	18–97 y	4021	Educational level (Illiterate, High school or less, University degree or higher)	OSA assessed by the self-reported Stop-Bang questionnaire which consists of 8 simple but efficient items for OSA screening, and respondents with 3 (or more) out of 8 items are scored positive.	The prevalence of high risk for OSA was significantly higher in less educated participants	According to the Stop-Bang questionnaire, almost 1 out of every 3 individuals were classified in the high-risk group for OSA Education did not have any significant effects on OSA in logistic multivariate regression

## Data Availability

No new data were created or analyzed in this study. Data sharing is not applicable to this article.
